# Scabies in Spain? A comprehensive epidemiological picture

**DOI:** 10.1371/journal.pone.0258780

**Published:** 2021-11-01

**Authors:** Lidia Redondo-Bravo, Beatriz Fernandez-Martinez, Diana Gómez-Barroso, Alin Gherasim, Montserrat García-Gómez, Agustín Benito, Zaida Herrador

**Affiliations:** 1 National Centre for Tropical Medicine, Health Institute Carlos III (ISCIII), Madrid, Spain; 2 National Centre for Epidemiology, Instituto de Salud Carlos III (ISCIII), Madrid, Spain; 3 Consortium for Biomedical Research in Epidemiology and Public Health (CIBERESP), Madrid, Spain; 4 Subdirección General de Sanidad Ambiental y Salud Laboral, Ministerio de Sanidad, Madrid, Spain; 5 Network Biomedical Research on Tropical Diseases (RICET in Spanish), Madrid, Spain; Fundacao Oswaldo Cruz, BRAZIL

## Abstract

**Introduction:**

Scabies is a neglected disease stablished worldwide with a fairy well determined incidence. In high-income countries, it often causes outbreaks affecting the residents and staff of institutions and long-term facilities, usually hard to detect and control due to the difficult diagnosis and notification delay. This study aim at characterizing the affected population, geographical distribution, and evolution of scabies in Spain from 1997–2019 as well as to describe the main environments of transmission using different data sources.

**Methods:**

We carried out a nationwide retrospective study using four databases, which record data from different perspectives: hospital admissions, patients attended at primary healthcare services, outbreaks, and occupational diseases. We described the main characteristics from each database and calculated annual incidences in order to evaluate temporal and geographical patterns. We also analyzed outbreaks and occupational settings to characterize the main transmission foci and applied Joinpoint regression models to detect trend changes.

**Results:**

The elderly was the most frequent collective among the hospital admitted patients and notified cases in outbreaks, while children and young adults were the most affected according to primary care databases. The majority of the outbreaks occurred in homes and nursing homes; however, the facilities with more cases per outbreak were military barracks, healthcare settings and nursing homes. Most occupational cases occurred also in healthcare and social services settings, being healthcare workers the most common affected professional group. We detected a decreasing trend in scabies admissions from 1997 to 2014 (annual percentage change -APC- = -11.2%) and an increasing trend from 2014 to 2017 (APC = 23.6%). Wide geographical differences were observed depending on the database explored.

**Discussion:**

An increasing trend in scabies admissions was observed in Spain since 2014, probably due to cutbacks in social services and healthcare in addition to worsen of living conditions as a result of the 2008 economic crisis, among other reasons. The main transmission foci were healthcare and social settings. Measures including enhancing epidemic studies and national registries, reinforcing clinical diagnosis and early detection of cases, hygiene improvements and training of the staff and wide implementation of scabies treatment (considering mass drug administration in institutions outbreaks) should be considered to reduce the impact of scabies among most vulnerable groups in Spain.

## Introduction

Scabies is a human ectoparasitosis produced by *Sarcoptes scabiei*, transmitted by direct contact with an infected person or less frequently through contaminated fomites if the host is infested with a large number of mites [1]. Classic infestation of scabies affects epidermis and manifestations include generalized and intense pruritus that worsens at night [2, 3].

Most common complications derive from its main symptoms (i.e. itch and scratch), which can lead to secondary infections by *S*.*pyogenes* and *S*.*aureus* [4], usually superficially localized at skin. Less frequently, scabies can cause severe complications, including invasive wound infections or at distance infections such as glomerulonephritis and rheumatic fever [5, 6]. Depending on the immune response and status, there is a severe form, known as Crusted or Norwegian scabies, in which the human host suffers a super-infestation by millions of mites. It is characterized by thick crusts of skin containing large numbers of scabies mites and eggs. This form usually relates to immunosuppression conditions, as advanced HIV, malignancy, or advanced age [7].

Scabies clinical presentation is usually unspecific, which may lead to a difficult and frequently delayed diagnosis or even misdiagnosis, and therefore difficulties in outbreaks management and control. First line treatment consists in topical use of permethrin, however oral use of ivermectin, which is not commercialized in some European countries as Spain, might be also considered [3], sometimes as mass drug administration (MDA) for community-guide control [8–10]. Although permethrin resistance has been pointed out as an emerging problem, the lack of treatment success could also be explained by incorrect use of permethrin or reinfestation due to incomplete management of surroundings [11].

The world distribution and extent to which scabies affects is unknown [8], Its occurrence is highly influenced by house living conditions (specially overcrowded dwellings) and other socioeconomic factors as poverty or lack of access to healthcare [12]. In high-income countries, scabies outbreaks are a challenging issue in nursing homes for the elderly and disabled people, schools, military facilities, and prisons since transmission to cohabitants is common. High attack rates have been described among staff working in these kind of facilities [13].

Scabies is rarely a mandatory reported disease, and even in high-income countries, it causes stigma, particularly in specific collectives. This, in addition to the absence of a unified registry or population-based studies makes difficult to estimate its incidence and magnitude.

The aim of this study is to characterize the affected population, geographical distribution, and evolution of scabies in Spain from 1997–2019 as well as to describe the main environments of transmission using different data sources. This would help understanding the epidemiological context of scabies in Spain and other high-income countries with similarities in terms of demography, healthcare, and social services; in order to allocate opportunely resources and orient targeted public health actions towards reducing the incidence and impact of this disease.

## Methods

### Study design and data collection

We carried out a retrospective cross-sectional study using four databases, which record health information for different purposes and from different settings/sectors/populations. CMBD (data from hospital admissions discharges), RENAVE (outbreaks data from the Spanish Surveillance Network), BDCAP (primary health care data of a representative sample of the Spanish population) and ODR (occupational diseases recorded and followed up by the National Social Security System).

#### CMBD

We used CMBD data from 1 January 1997 to 31 December 2017 (the available period). The CMBD database receives reports from ~98% of public and private hospitals. The International Classification of Diseases is used for codification. Ninth Revision, Clinical Modification (ICD-9-CM) and ICD-10-CM were the ICD versions in use during the study period. Registers with ICD-9-CM code 133.0 (‘Scabies’) and ICD-10 codes B86 (‘Scabies’) were analyzed. For each registry entry, main diagnosis (the major process considered as the primary reason for the patient’s hospital admission) and secondary diagnoses were analyzed; these can coexist with the main at the time of admission or may develop during the hospital stay. For each hospitalization, the variables collected were sex, age, date of admission, discharge type, autonomous region (AR) and province of residence, notifying AR, as well as other diagnoses codes that could affect immunity or affect scabies appearance risk (i.e., HIV, malignancy, osteoarticular and skin diseases for their relation to immunosuppression treatments, and treatment with immunosuppressive drugs, as described in S1 Table).

#### RENAVE

We obtained data for scabies outbreaks recorded from 1 January 2011 to 31 December 2019 (all data available) from the National Surveillance Network (RENAVE in Spanish) through a formal query to the National Center of Epidemiology of Spain. Records of each outbreak included: notifying AR, province and municipality where the outbreak occurred, number of people at risk of scabies transmission in the place of occurrence (close contacts identified in each outbreak), number of people with scabies, number of people admitted to hospital, number of deceased people, date of symptoms onset of the first case detected, date of symptoms onset of the last case detected and transmission-related setting/environment. Outbreaks attack rates and length of duration were calculated.

#### BDCAP

Data from -primary care was also requested for this study. BDCAP is a database which contains data from a representative sample at regional level of normalized clinical records from Primary Care. It takes into account the resident population assigned to a physician quota. It collects clinical information from a random sample of approximately 10% of all primary care medical records, including information on age, sex, AR of residence, rent, and employment status. We obtained all data available (from 2011–2017) with the diagnosis of scabies. Further information related to the sampling methodology is described elsewhere [14, 15].

#### ODR

Lastly, we used data from the Occupational Diseases Registry (ODR). Diseases are notified through an electronic application, created by the National Social Security System in 2007 to record information about professionals with an occupational disease. In this study we included all cases recorded with diagnosis of scabies codified according to CIE-10 (B86), from 2007–2019 (all data available). It also included age, sex, AR and province of occurrence, nationality, the year of notification, duration of the sick leave due to scabies, occupation according to the CNO classification (National Occupational Code) and exposure setting type -as economic activity according to CNAE93 and CNAE09 classifications (National Codes for Economic Activities)-.

These four databases are official and belong to the Spanish government (Ministry of Health and Ministry of Science) and are available for researchers access prior formal request (CMBD, ODR and RENAVE) or are open data (BDCAP). Information from all available years in each system were requested and all cases collected were included in the study.

Representativeness of the data differed according to the source, since they are designed for different purposes:

- CMBD includes only scabies severe cases since it collects all cases admitted to hospital. This is a national registry that includes 98% of all hospital admissions since 1997 and all AR are included.
- BDCAP includes a representative sample of all cases seen in Primary Care (10%of the cases, mild and severe). Some AR have included the data with a delay so time series assessment may be compromised.
- ODR only includes occupational scabies (for this reason age groups of <15 years old and 65 years and older are not represented). This is a national registry that includes all occupational diseases since 2007.
- RENAVE includes cases identified in outbreaks. It provides information regarding those groups among which outbreaks are more common and the extension of these outbreaks. However not all AR reported during the same period. For this reason, time series analyses may be compromised.

Official overall and active population figures from 1997 to 2019 for the Spanish AR and provinces were obtained from the Spanish Statistical Office (INE in Spanish) for rates calculations [16].

### Statistical analysis

As cases for each data set belonged to different time periods the annual average cumulative incidence was calculated for each dataset. We calculated scabies average annual cumulative incidences for each time period-database as the average number of annual scabies cases identified in each database / 106 inhabitants for CMBD, RENAVE and BDCAP, and /106 active population for ODR overall and by age groups. In addition, to describe the geographical distribution, we also calculated the average cumulative incidence for the period from 2011 until 2017 for each data source and (since those were the years present in all data sources) by AR. We performed Spearman´s Rho tests to assess the correlation between the average annual incidences of scabies according to each database by AR during that period in order to test the consistency of the geographical patterns present in each database.

For CMBD we also calculated the number of scabies admissions/10^6^ inhabitants per year by age groups and HIV status due to the relevance of immunosuppressive status and advanced age for severe scabies forms. To assess the time series evolution of scabies in Spain we used the CMBD data since the scabies admissions time series was the longest and the steadier due to the methodology applied in the data collection and codification. We applied Joinpoint regression models to detect trend changes in annual hospitalizations rates in HIV+ and non-HIV populations, applying Joinpoint Trend Analysis Software This technique provides estimates of annual percentage change (APC) in trends with corresponding 95% CIs. Seasonality was also assessed performing a regression analysis with the annual cumulative incidence of hospital admissions as a dependent variable and included trend and seasonality as independent variables by using sine and cosine functions.

We computed secondary attack rates from the outbreaks database as the number of scabies cases divided by the total people at risk in each outbreak (close contacts, household members or people working/living in the same closed setting) which were identified by public health officials from the different local public health authorities in each region, this information is available in the RENAVE database).

We used frequencies, percentages, mean ± standard deviation (SD), medians and interquartile range (IQR) to summarize data. Differences in proportions were assessed by the χ2 test with 95% confidence intervals (95% CIs). Student’s t-test was used to compare differences in the means. We performed non-parametric Kruskal-Wallis test for non-normal distributions and subsequent Man-Whitney U tests were employed in a post-hoc fashion to explain where amongst the independent groups the actual differences existed. We used two-sided tests, and P < 0.05 was considered significant.

Data were mapped using Flourish studio available at https://flourish.studio/.

Data analysis was performed using Stata version 15.0 and Joinpoint software version 4.2.0.1, National Cancer Institute, Bethesda, Maryland.

Data became available in different moments as different institutions provided the requested information. Each data base was explored and analyzed accordingly. Geographical distribution assessment using all data sources was performed once all the data had been received. Manuscript was drafted once all the analysis had been performed.

### Ethics statement

This study involves the use of patients’ medical data from the CMBD, BDCAP, RENAVE and ODR. CMBD and BDCAP data are hosted by the Ministry of Health (MoH). Researchers working in public and private institutions can request the CMBD database by filling in, signing and sending a request form and a confidentiality agreement—both available on the MoH website. The CMBD meets all the relevant legal and technical requirements as regards safe access and data protection. Formal ethical approval is not required for CMBD analyses. Not individualized BDCAP data is available for download in the virtual platform: https://pestadistico.inteligenciadegestion.mscbs.es/publicoSNS/S/base-de-datos-de-clinicos-de-atencion-primaria-. Formal queries to the National Center for Epidemiology for the RENAVE data and to the National Social Security System for ODR data, fulfilling the formal application and ethics requirements for this purpose were carried out. RENAVE registers anonymously information on notifiable diseases, following the mandate of Spanish and international regulations. All these databases meet all considerations regarding personal data protection.

## Results

### General description

We summarized the main characteristics found in the four databases in Table 1.

**Table 1:** General description of CMBD, RENAVE, BDCAP and CEPROSS databases.

CMBD 1997–2017				RENAVE 2011–2019				BDCAP 2011–2017				ODR 2007–2019			
N = 2.530	n°	freq.	Average Annual CI	N = 5.125	n°	freq.	Average Annual CI	N = 152.974	n°	freq.	Average Annual CI	N = 1.357	n°	freq.	Average Annual CI
**Overall**			3 (95%CI: 2–3)	**Overall**			13 (95%CI: 12–14)	**Overall**			488 (95%CI: 482–494)	**Overall**			2 (95%CI: 2–3)
**Age** (years)	**2530**			**Age**	**2604**	**50.8%**		**Age**	**152974**			**Age**	**1357**		
0–14	650	25.7%	5 (95%CI: 3–6)	0–14	452	8.8%	8 (95%CI: 6–10)	**0–14**	38040	24.9%	829 (95%CI: 806–851)	0–14	0	0.0%	0 (95%CI: 0–0)
15 a 24	182	7.2%	2 (95%CI: 1–3)	15 a 24	260	5.1%	6 (95%CI: 4–8)	15 a 24	24146	22.3%	666 (95%CI: 644–688)	15 a 24	92	6.8%	1 (95%CI: 0–2)
25 a 44	552	21.8%	2 (95%CI: 1–3)	25 a 44	512	10.0%	4 (95%CI: 3–5)	25 a 44	38960	25.9%	394 (95%CI: 384–405)	**25 a 44**	654	48.2%	4 (95%CI: 3–5)
45 a 64	403	15.9%	2 (95%CI: 1–2)	45 a 64	349	6.8%	3 (95%CI: 2–5)	45 a 64	34157	15.4%	439 (95%CI: 426–451)	**45 a 64**	607	44.7%	4 (95%CI: 3–5)
**≥65**	743	29.4%	5 (95%CI: 3–6)	**≥65**	1031	20.1%	15 (95%CI: 12–17)	≥65	17671	11.6%	324 (95%CI: 311–336)	≥65	0	0.0%	0 (95%CI: 0–0)
				**ND** (no data)	**2521**	**49.2%**									
**Sex**	**2530**			**Sex**	**2387**	**46.6%**		**Sex**	**152974**			**Sex**	**1357**		
Female	1064	58.0%		Female	1475	51.8%		Female	74147	48.5%		Female	1083	79.8%	
Male	1466	42.0%		Male	912	38.2%		Male	78827	51.5%		Male	274	20.2%	
				**ND**	**2738**	**53.4%**									
**Discharge**				**Hospitalized**	49	1.0%		**Rent** ^b^	**145751**			**Exposure setting type**			
Home	2393	89.7%		**Deaths**	2	0.0%		≥100.000 €/yr	0	0.0%		Healthcare	475	35.0%	
Death	85	3.2%		**Outbreaks attack rate** (%)	mean	median		18.000–99.999 €/yr	19093	13.0%		Social services	527	38.8%	
Others	189	7.1%			39.7	26.7		<18.000 €/yr	107471	73.2%		General administration	263	19.4%	
					min	max		Very low^c^	18798	12.8%		Education & Public order	22	1.6%	
					0.4	100		Unclassified	389	0.3%		Others	70	5.2%	
**Comorbidities**				**Outbreaks length** (in days)	mean	sd		**Employment status** ^b^	**146580**			**Work leave duration**	mean	sd	
HIV+	274	10.8%			59.9	79.6		Active	40238	27.4%			6.1	20.8	
Neoplasms	171	6.8%			p25	8		Unemployed	16205	11.0%			p25	0	
Skin and subcutaneous tissue	394	15.6%			p50	32		Non-active	63731	43.4%			p50	0	
Musculoskeletal.s /connective t.	163	6.4%			p75	77		Pensioner	22911	15.6%			p75	7	
Immunosuppressive drugs	10	0.4%						Other status	3495	2.4%					

^a^Average Annual CI (Cumulative Incidence): Average number of scabies cases identified annually in each data source per million inhabitants (CMBD.RENAVE.BDCAP) /per million active population (ODR)

^b^Rent and employment status data are available from 2012 (in 2011 n = 6.093 unclassified)

^c^Very low refers to people earning money by other activities different from work in a very precarious context.

Different characteristics were present in each database according to their purpose:

#### CMBD

The average annual cumulative incidence was 3 scabies hospital admissions per million inhabitants. The age groups with highest annual cumulative incidence were those of 0–14 years old and 65 years and older. Cases occurred with a frequency slightly higher in women (58%).

From all admitted cases, nearly 90% were discharged home. Only 3% died. Most frequent scabies-related comorbidities were those affecting skin and subcutaneous tissues (16%) and HIV (11%).

#### RENAVE (outbreaks)

A total of 672 outbreaks were reported to the RENAVE from 2011 to 2019 with 5,125 cases and an average of 8 cases per outbreak. The average annual cumulative incidence was 13 cases identified in outbreaks per million inhabitants. The highest annual cumulative incidence was registered in the age group of 65 and older. Cases occurred with a frequency roughly equal in women and men. From cases reported in outbreaks, less than 1% needed hospitalization, and less than 0.05% died. The outbreaks attacks rates when the information was present ranged from 0.4% to 100%, and the median length of the outbreaks was 32 days (IQR 8–77).

**Fig 1:**
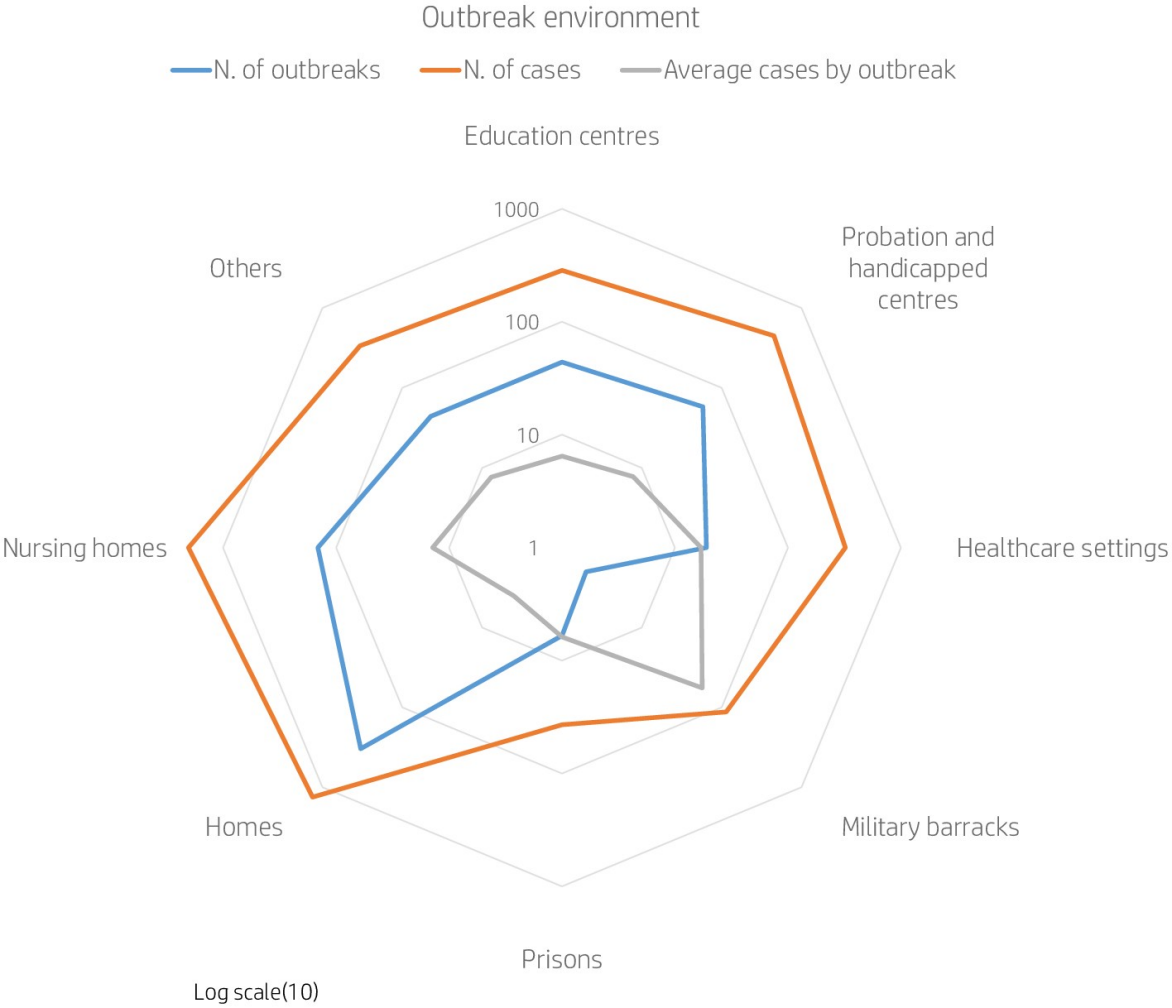
No. of outbreaks, no. of cases and average cases per outbreak by setting/place of occurrence by RENAVE outbreaks data.

We show a general description by environment of transmission in Table 2 and Fig 1 (this information was available for 95% of the outbreaks). Most reported outbreaks occurred in homes and nursing homes; however, the outbreaks with higher number of cases took place in military barracks, followed by healthcare settings and nursing homes.

**Table 2:** Number of outbreaks declared to RENAVE, cases and average cases by outbreak place of occurrence, from 2001 until 2019 in Spain.

Settings and other places where outbreaks occurred	N. of outbreaks	%	N. of cases	%	Average cases by outbreak
Education centres	44	6.5%	285	5.6%	6
Probation and handicapped centres	58	8.6%	450	8.8%	8
Healthcare settings	19	2.8%	323	6.3%	17
Military barracks	2	0.3%	114	2.2%	57
Prisons	6	0.9%	37	0.7%	6
Homes	330	49.1%	1326	25.9%	4
Nursing homes	145	21.6%	2029	39.6%	14
Others	44	6.5%	337	6.6%	8
Unknown	24	3.6%	224	4.4%	9
**Total**	**672**		**5125**		**8**

**Fig 2:**
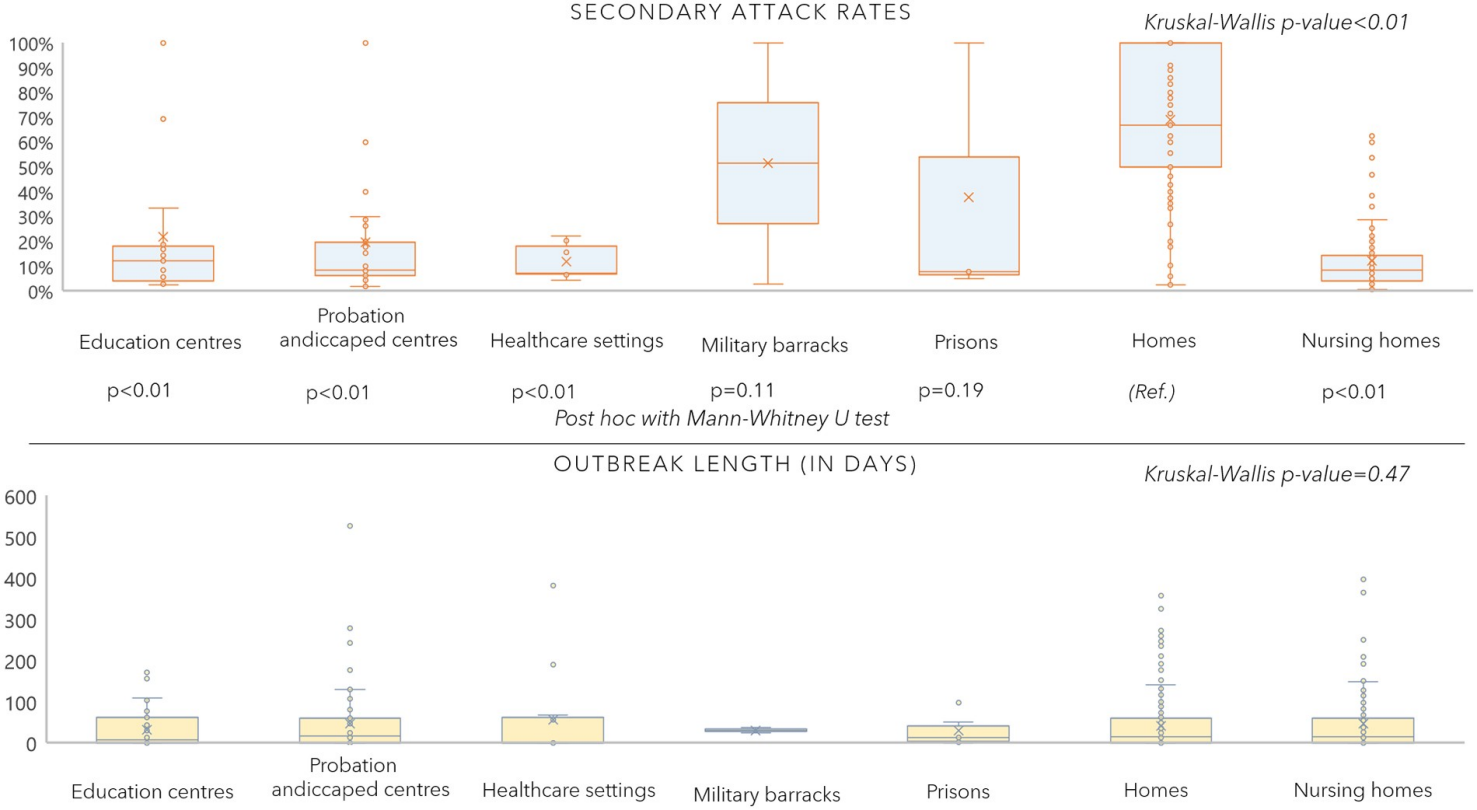
Secondary attack rates and outbreaks length by setting/place of occurrence.

We compared the outbreaks attack rates and length (in days) by setting and place where they took place. We found statistically significant differences in attack rates (Fig 2). Homes, prisons, and military barracks showed the highest rates. However, we found no differences in the length of the outbreaks by environment of transmission.

#### BDCAP

The average annual cumulative incidence was 488 scabies cases seen in Primary Care per million inhabitants. The highest annual cumulative incidences were registered in the youngest age groups (especially younger than 15 years old). Cases occurred with a frequency roughly equal in women and men. Most cases seeking attention in Primary Care settings earnt less than 18.000 euros/year (73.2%) and almost half of them were non-active population (understood as those people not working, not registered as pensioner nor unemployed, as children or individuals beneficiaries of a social security holder).

#### ODR

The average annual cumulative incidence was 2 occupational scabies cases per million active population. The highest annual cumulative incidences were registered in the age groups of 15–24 and 45–64 years old. Most cases occurred among females (roughly 80%) and in social services (38%) and healthcare (35%) settings. 5% of these cases were not Spanish. The median of the work leave duration was 6.1 ± 20.8 days.

**Fig 3:**
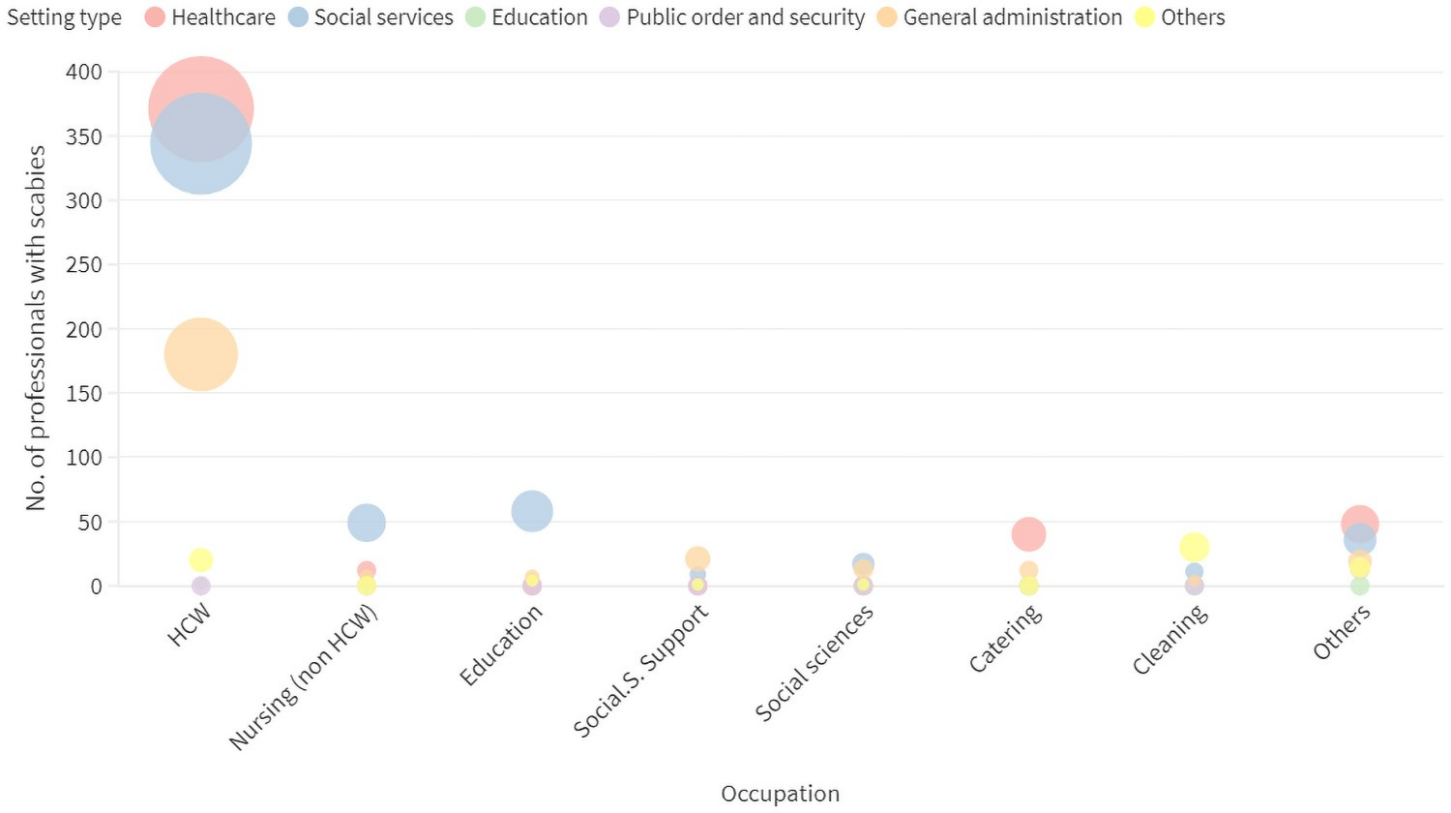
Work-related scabies cases by setting type and occupation. Dot´s size represents the number work-related scabies cases. HCW: Healthcare workers.

According to occupations distribution (Fig 3) healthcare workers were the most affected professionals (Chi^2^ p-value <0.001). For this group, transmission occurred in both healthcare and social services settings. Social services settings included mostly residences and centers for nursing old and disabled people requiring healthcare services. The other two most affected professional groups were those related with home nursing, education and social support carried out mostly in social services settings (Fig 3).

### Scabies admissions time series (overall, by HIV status and age groups)

Joinpoint analyses showed a decreasing trend in patients admitted to hospital with scabies until 2014 when it started to increase (annual percentage change -APC: from 1997–2014:-11.2% (-12.7%: -9.7%); APC from 2014–2017: 23.6% (-2.9%:57.2%). Seasonality was also found, showing a peak during the winter months (highest rates in January and a marked decreased during the summer (with lowest rates in July).

**Fig 4:**
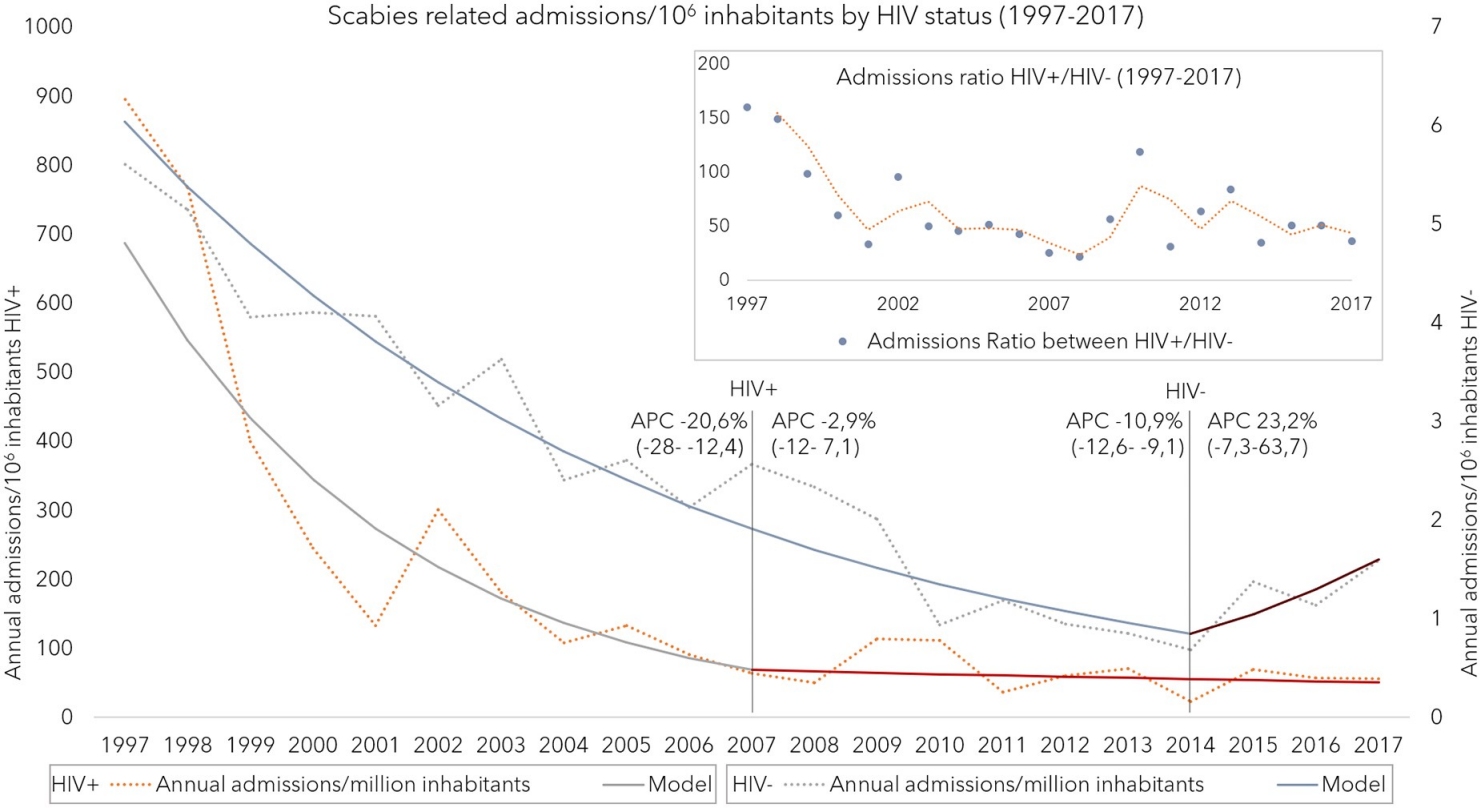
Scabies annual admissions joinpoint regression model by HIV status and HIV+/HIV- scabies admissions ratio, from 1997–2017 in Spain. APC: Annual percentage change.

When analyzing temporal trends according to HIV status, we detected in non-HIV an APC of -10.9% until 2014 when the APC changed to 23.2%. However, in HIV+ the observed decreasing trend showed a -20.6% APC until 2007 when the APC became 2.9%. The rates ratio of scabies admissions between HIV+ and non-HIV varied from a maximum of 128 in 1997 to a minimum of 21 in 2008. This ratio shows a sharped decreasing trend until 2001, followed by a soft decreasing trend until 2008 when increases were observed. However, we did not find statistically significant joinpoints (Fig 4).

Joinpoint regression analyses performed by age groups showed changes in the decreasing trends in all groups except for ≥65. However, these joinpoints took place in different years: 2013 (15–29 and 50–64 age groups), 2014 (0–14 age group) and 2015 (30–49 age group).

### Geographical pattern

**Fig 5:**
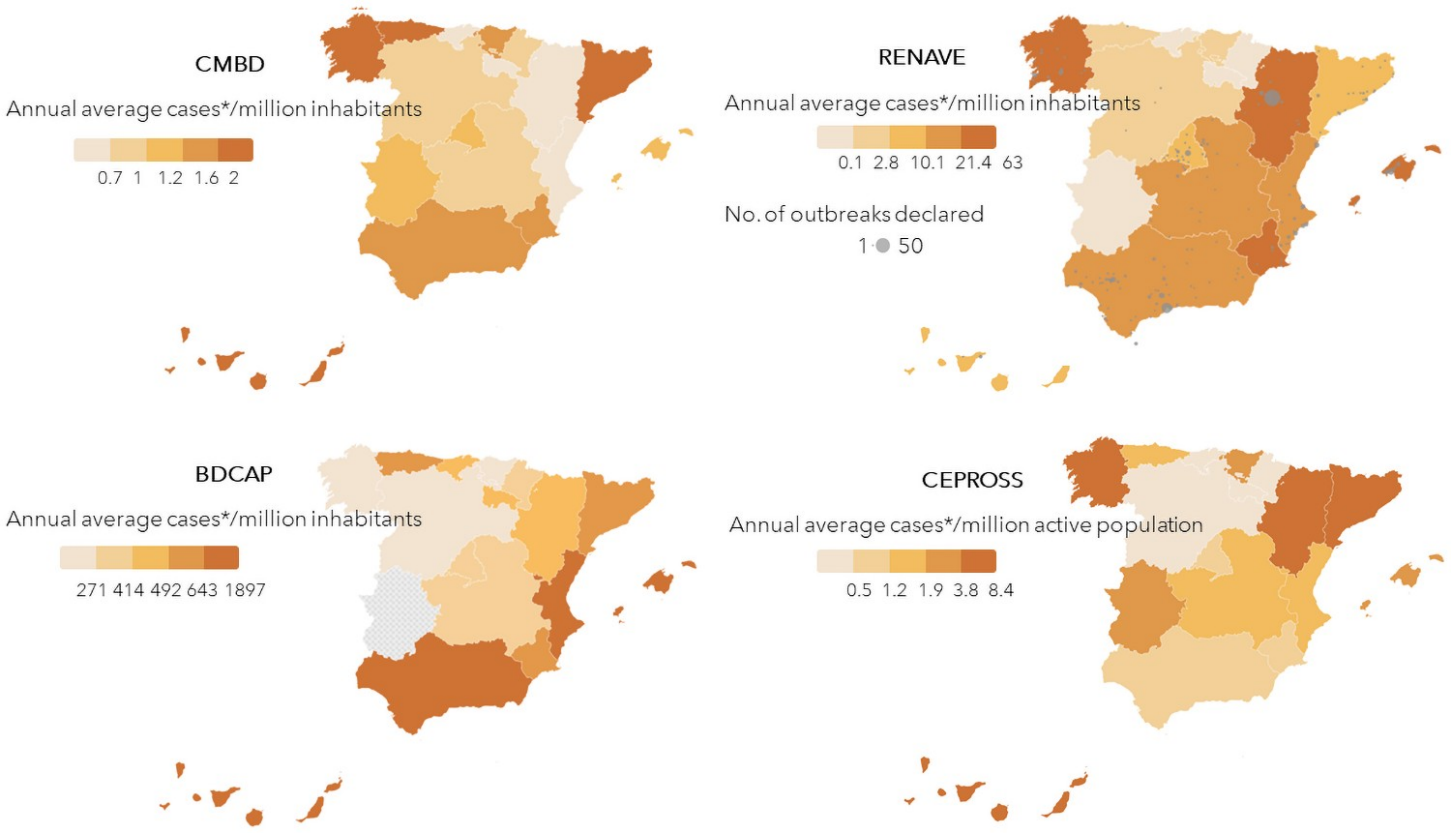
Annual average scabies cases/million inhabitants, from 2011–2017 in Spain by CMBD, RENAVE (outbreaks), BDCAP and ODR. *Cases according to each database: CMBD: Scabies related admissions/million inhabitants. RENAVE: Scabies cases detected in outbreaks/million inhabitants. BDCAP: Scabies cases diagnosed in Primary Care/million inhabitants. ODR: Occupational scabies cases/million active population.

The AR with highest scabies incidences according to each information source are described hereafter (Fig 5) (Spanish Autonomous Regions are shown in S1 Fig).

The AR with highest incidences during the period from 2011 to 2017 were Asturias, Aragon and Galicia (according to CMBD); Canary Islands, Balearic Islands and Valencian Community (according to BDCAP); Balearic Islands, Aragon and Galicia (according to RENAVE), being the AR with more outbreaks notified Andalusia, Aragon and Valencian Community. Finally, according to ODR, during that period the AR with the highest incidences of scabies as an occupational disease were Catalonia, Galicia and Aragón (S2 Table).

**Fig 6:**
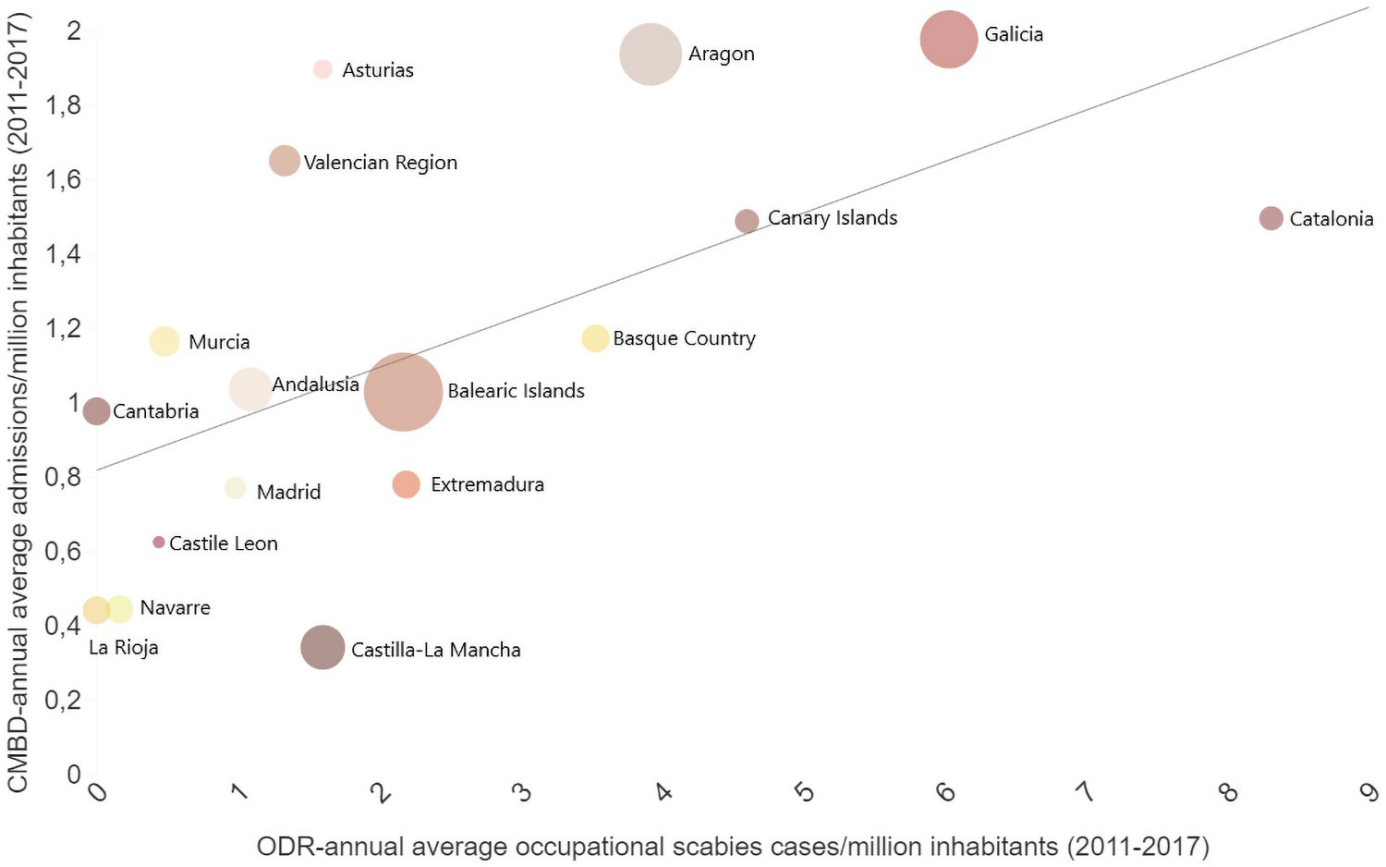


Rho Spearman´s test results for incidence correlation by AR among the four information sources are shown in Table 3. We only found a positive correlation between CMBD and ODR incidence data from 2011–2017 (Fig 6).

**Table 3:** Spearman´s Rho correlation test between all scabies databases combinations by AA.CC, from 2011 to 2017 in Spain.

Databases	Spearman´s Rho	p-value
CMBD-ODR	0.66	**0.004**
ODR-RENAVE	0.42	0.10
CMBD-BDCAP	0.23	0.38
CMBD-RENAVE	0.45	0.07
RENAVE-BDCAP	0.27	0.31
CEPROSS-BDCAP	0.13	0.62

## Discussion

This is the first nationwide study about scabies in this country. Few papers have been published regarding this topic and most of them are focused on case studies and outbreaks description and management [17–21]. We present an innovative approach using four national data sources, which record health related information from different perspectives, and for different purposes (hospital admissions, outbreaks surveillance, primary care attention and occupational diseases surveillance). This integrated information leads to obtain a wider vision of the epidemiologic situation of scabies in Spain to allocate resources more efficiently, and it might be of help for other countries with similar contexts. Our findings also reflect the need of a national homogeneous registry of which according to scabies admissions data seems a growing health concern in Spain.

### General characterization

As for age groups distribution of scabies, in both CMBD (hospital admissions) and RENAVE (outbreaks) above 65 years old was the most represented age group. The explanation is probably that, in one hand, aging is a risk factor for crusted scabies, due to immunosenescence, and therefore the risk of needing hospital admission is higher [7]. On the other hand, many of the reported outbreaks in western countries occur in elderly nursing homes, so is not surprising finding ≥65 as the most affected group according to our outbreak’s surveillance information [22–24].

However, according to the Primary Health Care data, children (0–14) young adults (15–24) and were the most affected. These age groups have usually less risk of suffering severe scabies forms due to an adequate immune response and are therefore less represented among those admitted to hospital. Wide differences were observed in the values of the annual cumulative incidences according to primary care and the rest of the data sources (hospital admissions, outbreaks investigations and occupational environments) from these figures we can conclude that transmission in the community is high and must be taken into consideration too when updating public health strategies for scabies.

Finally, we must consider that each database has a different purpose, for instance ODR is meant to register occupational diseases so it just includes active population excluding elderly and children.

Children, adolescents, and elderly have been pointed out by other studies as the most affected groups worldwide [8]. However, for western countries the differences among age groups are narrower than in low-income countries [8]. This matches our findings, which means that using different data sources can provide a wide and solid information and feasible explanations in this connection.

With respect to sex, as in previous studies there was an even distribution in all data sources except for ODR [8]. This is probably explained by the high representation of women among HCW and other caregiver professionals [25].

Other frequent characteristics present among these patients were low (<18 000 €) and very low rental incomes and being part of non-active population. It highlights that, in Spain it seems to be a neglected disease too as it might be in other high-income countries, and as such, vulnerable population with low socioeconomic status are those at highest risk [26].

In regards of concomitant health conditions, skin and subcutaneous tissue diseases were the most common among those admitted to hospital followed by HIV. While HIV has been previously described as a risk factor for crusted scabies [27], skin lesions cannot be established as a such, since they are also a consequence of the scabies infestation (most frequent complications are localized secondary infections by *S*.*pyogene*s and *S*.*aureus* caused by scratching) [28]. In both CMBD and RENAVE, fatality rates were low (although death was not necessarily attributable to scabies since the cause of death is not recorded in CMBD or RENAVE database). However, scabies has been related more often with long-term complications as glomerulonephritis or rheumatic fever that can reach fatality rates of 5–10%/year [5, 6, 28]. Although in our database the percentage of patients admitted with scabies and glomerulonephritis or rheumatic fever was very low (less than 1%) it might be explained by the delay between scabies infestation and invasive complications.

### Outbreaks, settings, and occupational transmission

Our study also provides a wide perspective on main affected settings since we analyzed both outbreaks and occupational data sources. Nursing homes and social services institutions were pointed as high-risk facilities for residents and staff, as previously mentioned in the literature [13, 22]. However, according to outbreaks-related data, homes were the most frequent environments of transmission with also high attack rates, which agrees with the cumulative incidence observed in Primary Care pointing out that a wide amount of the burden of scabies nowadays is also community acquired. This highlights the need of national diseases registries which may provide a clearer image of the burden of disease for scabies as well as other diseases not included in the current surveillance systems. Nevertheless, due to the reduced number of residents in homes, the number of cases/outbreaks were low in comparison with military barracks, health care settings and nursing homes. It seems feasible to think that the origin of these clusters might have been outbreaks affecting other settings as nursing homes or social services facilities where a household member was exposed. Occupational exposition may have a main role, probably related with health and social care activities, since the most affected professionals according to ODR were HCW from healthcare and social services. Even in hospitals, index cases have been also related with outbreaks in nursing homes [18]. It results challenging since residents living in these institutions often suffer dementia or other cognitive disorders, which contribute to a delay in the diagnosis and the implementation of measures for outbreaks control [22]. In our study the median length since the symptoms onset of the first case to the last one was around a month, albeit in some cases outbreaks lasted more than a year. In fact, scabies has been pointed out as one of the pathogens causing longest outbreaks in institutions [13].

### Time series

Time series analyses show a change in the decreasing trend of scabies admissions observed since the late 90´s. From 1990–2015 in Western Europe a decreasing mean percentage change of 0.32% has been described for scabies DALYs, albeit, it has been proven in North America an increase of 24% [8]. In Spain, we observed a change in the trend, with an increase since 2014. However, among HIV positive population, a change in the decreasing trend was detected earlier, in 2007.

The explanation of the change in trend in 2014 is not easy and it is probably multifactorial. However, the economic crisis may have played a main role, as it resulted in budget and social cutbacks, which had an impact in social institutions and the healthcare system [29–31]. The AROPE index (which reflects the percentage of people at risk of social exclusion) shows an increase in Spain since 2008 that peaked in 2014. This may indirectly reflect the impact of the crisis in living conditions [32]. The early change in HIV positive admissions rates (2007) could be explained by the effect of the economic crisis in population movements. According to the National Institute of Statistics (INE) in 2008, a substantial drop was observed in migrant population living in Spain and values before 2008 had not been reached yet in 2017 [33]. As the HIV prevalence in migrants is higher than in local population and some migrant groups might be particularly vulnerable for scabies due to living conditions [34], a possible explanation would be that the decrease in migrants would follow a stabilization in HIV positive admissions trends due to the worsening living conditions of the remaining ones. In addition, when analyzing the evolution of the scabies admissions ratio between HIV+/HIV- population in Spain, we can also observe a decrease in the gap between these groups that became wider again during the economic crisis (2008–2014). However, we must consider that changes in the HIV treatment and its access may have also played an important role among HIV positives population during this period. These results highlight the increasing incidence of scabies in Spain, which might be explained by a worsening in living conditions probably exacerbated in social and healthcare settings as part of the 2008 economic crisis impact.

Lastly, it should be also taken into account that in some countries changes in the use of different therapeutics due to their authorization played a role in the incidence changes observed. However, in Spain permethrin was authorized since 2000 and recommended as first line treatment [35–37].

Regarding the seasonality detected, it agrees with studies relating scabies risk with cold temperature and humidity [38].

### Geographical pattern

Depending on the data source, different regions of Spain were the most affected being hard to establish a clear pattern. However, as the outcomes measured are not the same (admissions, outbreak-related cases, cases detected in primary care and occupational cases), the differences observed in the scabies spread might be explained by demographical patterns (i.e. presence of institutionalized elderly people, immigrants living in poor conditions or people at risk of social exclusion). However, one of the main reasons why we only found a significant positive correlation between CMBD and ODR may be related with the reporting bias by AR in the other datasets.

### Limitations

As previously mentioned, the main limitation is the lack of representation of some AR due to delays and under-reporting in some regions. This affected more BDCAP and outbreaks declared in RENAVE. It may lead to a reporting bias when analyzing temporal trends and geographical spread. CMBD and ODR are those with the most reliable information in terms of time and distribution since the notification is mandatory and opportunely made. For this reason, for time series analyses we decided to use the most stable and complete data source (CMBD) which was also the longest series. Although it only represents hospital admissions and therefore severe cases, scabies admissions incidence may act as a proxy for global incidence (nevertheless, although these are probably the most severe forms, there is no codification for crusted scabies, so we couldn’t perform a stratified analysis). However, although the CMBD has maintained its collection process and uses the same classification for codification (the ICD9 from 1997–2015 and ICD10 since 2016) it must be taken into account that changes in the clinical practice, diagnosis techniques or health-seeking behavior may have varied along time affecting the evolution of scabies admissions and may also differ by regions, explaining partially the geographical distribution of scabies in Spain. ODR albeit being a complete database it only reflects occupational exposure and risks. For all these reasons, we decided to geographically represent all the data sources and compare its distributions to assess their consistency to better understand the geographical pattern of scabies in Spain in terms of admissions, declared outbreaks, occupational transmissions and primary care seeking attention. We must also consider that demographic differences in the population pyramid by regions could also play a role in some of the geographical differences observed.

## Conclusions

Scabies is considered a neglected disease with a considerable burden of disease around the world [8], even in high income countries as we show in this study. Research interest has recently increased in diagnosis treatment and population-based interventions. In this study we highlight the rising incidence of scabies in Spain (using the most steady and representative data source), which has been also observed in other high-income countries [8]. Vulnerable collectives as children, the elderly, institutionalized population and individuals with low incomes are the most affected. It seems to have also acquired an important role among professionals, and especially those related to healthcare and social services. The need of preventive measures implementations in this area is remarked and probably related with resources cutbacks. However challenges in managing scabies outbreaks as late diagnosis and recognition of outbreaks, logistically difficult mass treatment, as distressing treatment process may result in high costs [22]. Contact precautions, linen and material cleaning and disinfection, among other hygiene measures, should be reinforced in healthcare settings and institutions, including training sessions among the staff. Nevertheless, although clinical diagnosis should be reinforced among clinicians, overdiagnosis and overtreatment may also exist, particularly in outbreak settings. Therefore, clinical consensus, medical guidelines and training is also needed. We have observed the highest cumulative incidences in Primary Care, so primary care physicians should be a key stone in this strategy.

We used four different sources of information due to the lack of a representative data source/registry at national level. As we observed, only those databases with mandatory reporting were consistent among them regarding geographical distribution, bringing to light the need of implementing this kind of strategies in order to obtain unbiased epidemiologic information. As scabies is considered a truly neglected disease it is largely absent from global agenda which is essential to stablish an accurate estimate of its real burden [28]. In this sense, unless neglected diseases are included in nationwide registries and the existing outbreaks notification is improved (maybe by digitalization of clinical records and its integration with surveillance systems), the real estimates would be hard to obtain.

Among the steps towards control, one of the initial priorities, as we just mentioned, is to enhance clinical and epidemiologic study to better understand the burden of disease [28]. Scabies drug resistance must be surveilled too as it is an emerging concern [11]. In addition, we would like to underline the need of reinforcing the implementation of prevention measures, early detection, and control, especially in healthcare settings and social institutions. These should comprise: inclusion of scabies in differential diagnosis of dermatological diseases in institutionalized patients, reinforcement of contact precautions when a case is identified, training for social and health services staff, implementation of scabies treatment for all household members and other potentially exposed persons to prevent possible reexposure and reinfestation, and consideration of treatment with ivermectin in closed institutions outbreaks. The lack of its opportunely implementation because of a low resources investment may result in the long term in higher economic costs and substantial impact within the most vulnerable collectives.

## Supporting information

S1 Fig Spain autonomous regions map. (TIF)

S1 Table Diseases and other health conditions associated with scabies and their respective ICD-9 and ICD-10 codes. (DOCX)

S2 Table Autonomous regions with highest incidences of scabies according to CMBD, RENAVE, BDCAP, and ODR, from 2011 to 2017 in Spain. (DOCX)

S1 Checklist (DOC)
