# Supplementary figures and images for: Scabies in Spain? A comprehensive epidemiological picture
Source: PLoS One. 2021 Nov 1;16(11):e0258780. doi: 10.1371/journal.pone.0258780 (PMC8559925; doi:10.1371/journal.pone.0258780)

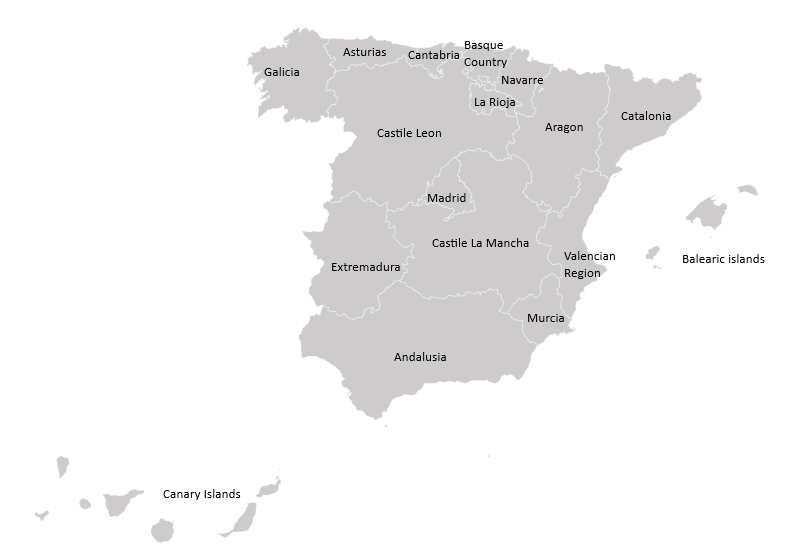

Supplement: S1 Fig — (TIF) [file pone.0258780.s001.tif]
