# Supplementary material for: Scabies in Spain? A comprehensive epidemiological picture
Source: PLoS One. 2021 Nov 1;16(11):e0258780. doi: 10.1371/journal.pone.0258780 (PMC8559925; doi:10.1371/journal.pone.0258780)
Supplement: S1 Table — (DOCX) [file pone.0258780.s002.docx]

**S1 Table. Diseases and other health conditions associated with scabies and their respective ICD-9 and ICD-10 codes.**

| **Diseases and other health conditions** | **ICD-9 codes** | **ICD-10 codes** |
| --- | --- | --- |
| HIV | 042, V08 | B20 |
| Neoplasms | 140-239 | C00-D49 |
| Skin and subcutaneous tissue | 710-739 | M00-M99 |
| Musculoskeletal system and connective tissues | 680-709 | L00-L99 |
| Immunosuppressive drugs | 963.1, E.858.1, E.933.1, E950.4, E962.0, E980.4 | T45.1 |
