# Supplementary material for: Scabies in Spain? A comprehensive epidemiological picture
Source: PLoS One. 2021 Nov 1;16(11):e0258780. doi: 10.1371/journal.pone.0258780 (PMC8559925; doi:10.1371/journal.pone.0258780)
Supplement: S2 Table — (DOCX) [file pone.0258780.s003.docx]

**S2 Table. Autonomous regions with highest incidences of scabies according to CMBD, RENAVE, BDCAP, and ODR, from 2011 to 2017 in Spain.**

| **Data source** | **AR** | **Incidence** |
| --- | --- | --- |
| CMBD | Asturias | 2 annual scabies admissions/10^6^ inhabitants |
|  | Aragon | 2 annual scabies admissions/10^6^ inhabitants |
|  | Galicia | 2 annual scabies admissions/10^6^ inhabitants |
| RENAVE | Balearic Islands | 63 scabies annual cases/10^6^ inhabitants |
|  | Aragon | 35 scabies annual cases/10^6^ inhabitants |
|  | Galicia | 26 scabies annual cases/10^6^ inhabitants |
| BDCAP | Canary Islands | 1898 scabies annual cases/10^6^ inhabitants |
|  | Balearic Islands | 1466 scabies annual cases/10^6^ inhabitants |
|  | Valencian Community | 939 scabies annual cases/10^6^ inhabitants |
| ODR | Catalonia | 8 annual occupational scabies cases/10^6^ active population |
|  | Galicia | 6 annual occupational scabies cases/10^6^ active population |
|  | Aragon | 4 annual occupational scabies cases/10^6^ active population |
